# Body Composition in Crohn's Disease and Ulcerative Colitis: Correlation with Disease Severity and Duration

**DOI:** 10.1155/2017/1215035

**Published:** 2017-10-31

**Authors:** Dawesh P. Yadav, Saurabh Kedia, Kumble Seetharama Madhusudhan, Sawan Bopanna, Sandeep Goyal, Saransh Jain, Naval K. Vikram, Raju Sharma, Govind K. Makharia, Vineet Ahuja

**Affiliations:** ^1^Department of Gastroenterology and Human Nutrition, All India Institute of Medical Sciences, New Delhi 110029, India; ^2^Department of Radiodiagnosis, All India Institute of Medical Sciences, New Delhi 110029, India; ^3^Department of Medicine, All India Institute of Medical Sciences, New Delhi 110029, India

## Abstract

**Background:**

Results on body composition in Crohn's disease (CD) and ulcerative colitis (UC) have been heterogeneous and are lacking from Asia. Present study assessed body composition in CD/UC and correlated it with disease severity/duration.

**Methods:**

Patients of CD/UC following between Dec 2014 and Dec 2015 who consented for bioimpedance analysis for body fat measurement were included. Lean mass and fat-free mass index (FFMI) were calculated with standard formulae. Visceral fat area (VFA), subcutaneous fat area (SCA), and visceral to subcutaneous fat ratio (VF/SC) were evaluated in CD patients on abdominal CT.

**Results:**

Lean mass in CD (*n* = 44, mean age: 41.2 ± 15.8 years, 73% males) was significantly lower than UC (*n* = 53, mean age: 33.2 ± 11.2 years, 68% males; 44.2 ± 7.8 versus 48.3 ± 8.4 Kg, *p* = 0.01). In both UC/CD, disease severity was associated with nonsignificant decline in BMI (UC: 22.1 ± 4.9 versus 20.2 ± 3.2 versus 19.9 ± 3.2 kg/m^2^, *p* = 0.23; CD: 22.1 ± 4.2 versus 19.9 ± 2.3 versus 19.7 ± 4.2 kg/m^2^, *p* = 0.18) and fat mass (UC: 10.9 ± 8.9 versus 8.1 ± 5.9 versus 5.7 ± 3.6 kg, *p* = 0.14; CD: 11.2 ± 7 versus 7.9 ± 4.4 versus 7.2 ± 5.9 kg, *p* = 0.16), and disease duration was associated with significant decline in FFMI (*p* < 0.05). In CD, disease severity was associated with nonsignificant decline in SCA and increase in VF/SC.

**Conclusions:**

CD patients have lower lean mass than UC. Body fat decreases with increasing disease severity and fat-free mass decreases with increasing disease duration in both UC/CD.

## 1. Introduction

There has been a recent rise in the disease burden of inflammatory bowel disease (IBD) in India and other Asian countries and as per a recent report, the overall number of IBD patients in India is second highest in the world after USA [1–3]. This increase will gradually burden the healthcare system and will put increasing pressure on the IBD physician for optimal care of patients. The optimal IBD care dose not only include the control of disease activity with immune based therapies, but also include improvement in quality of life, part of which is compromised by poor nutritional status of the patient which may be caused by low dietary intake, changes in metabolism, increased intestinal protein loss, and nutrient malabsorption [4]. The conventional indices for assessment of nutritional status such as body mass index (BMI) have been suboptimal and therefore require better modalities such as fat mass and fat percentage (for assessment of body fat) and fat-free mass index (FFMI) and lean mass (for assessment of fat-free mass) which can be assessed by bioimpedance analysis or DEXA (dual energy X-Rat absorptiometry) [5, 6]. There are several recent reports which have assessed and compared body composition with these techniques in patients with Ulcerative colitis (UC) as well as Crohn's disease (CD), but the results from these studies have been heterogeneous and inconsistent and very few of these studies are from Asian countries [7–11]. The distribution of body fat is different between Asians and Caucasians and therefore the results from West cannot be extrapolated to Asian patients. Abdominal fat (visceral and subcutaneous fat and visceral to subcutaneous fat ratio) has also studied in IBD and studies have inconsistently linked visceral fat to severity of CD and postoperative recurrence of CD [12–15].

This study was therefore planned to compare body composition between patients of UC and CD, correlate body composition with disease severity and duration in both UC and CD patients, correlate body composition with disease behavior in CD and disease extent in UC, and correlate abdominal fat with disease behavior, severity, and disease duration in CD patients.

## 2. Materials and Methods

### 2.1. Patient Population

Adult patients with diagnosis of ulcerative colitis and Crohn's disease attending the inflammatory bowel diseases (IBD) clinic at All India Institute of Medical Sciences, New Delhi, India, from Dec 2014 to Dec 2015, were screened for inclusion in the study. Of these, patients who consented for bioimpedance analysis were included in the study. Written informed consent was obtained from all patients included in the study. Institutional ethics committee approved the study.

### 2.2. Study Design

In this prospective study, patients with UC and CD were subjected to a uniform clinical evaluation including detailed history and examination, laboratory assessment, endoscopy (UGI endoscopy or colonoscopy as appropriate), and mucosal biopsies. Patient data was collected on their demographics, clinical, endoscopic, histologic, and radiologic features and treatment given and its response. All patients underwent body fat measurement with the bioimpedance machine. In addition, visceral fat area, subcutaneous fat area, and visceral to subcutaneous fat ratio (VF/SC) was evaluated in patients with Crohn's disease with the help of abdominal CT.

### 2.3. Definitions

#### 2.3.1. Crohn's Disease

The patients were diagnosed as CD on the basis of the European Crohn's and Colitis Organization (ECCO) guidelines, with a combination of clinical, endoscopic, and histological features [16]. The location and behavior of Crohn's disease were classified as per Montreal classification [17]. Severity of CD was measured by Crohn's disease activity index (CDAI) [18].

#### 2.3.2. Ulcerative Colitis

Diagnosis of ulcerative colitis was made on the basis of European Crohn's and Colitis Organization (ECCO) guidelines with a combination of clinical symptoms like bloody diarrhoea and endoscopic appearance and histological findings compatible with ulcerative colitis [19]. Disease activity in UC was measured by Simple Clinical Colitis Activity Index [20] and endoscopic severity was assessed by Baron's endoscopic score [21].

#### 2.3.3. Technique of Measuring Total Body Fat

Total body fat was measured by leg to leg bioimpedance machine (TANITA body composition analyzer, model TBF-215) in patients of ulcerative colitis and Crohn's disease [22]. Subjects were asked to stand barefoot on the metal sole plates of the machine, and age, gender, and height details were entered manually into the system via a keyboard. Body weight and percentage body fat, estimated using the standard built in prediction equations for adults, were displayed on the machine and printed out.

#### 2.3.4. Technique of Measuring Lean Mass and Fat-Free Mass Index

Lean mass was calculated with the following formula:

  Lean mass = Body weight *∗* (1 − (body  fat%/100)).

Fat-free mass index (FFMI) was calculated with the following formula:

  FFMI = (Lean mass/2.2)/((Height in meters)^2^  × 2.20462).

#### 2.3.5. Technique of Measuring Visceral Fat

Visceral fat (VF) and subcutaneous fat (SC) area were measured at the level of umbilicus (L4 vertebrae level) with the patient in supine position by multislice CT scanner (Siemens, Erlangen, Germany) in all the patients. The technique used for fat tissue measurements on CT has been standardized and validated [23]. The VF area was defined as all the pixels with adipose tissue attenuation coefficients. The tomographic attenuation of adipose tissue was defined to be between −150 and −50 Hounsfield units. The CT measurement was done by a radiologist blinded to the clinical data.

### 2.4. Statistical Analysis

Qualitative data was expressed as frequency and percentage. Quantitative data was expressed as mean ± SD if normally distributed and as median (interquartile range) when the data distribution was skewed. BMI, fat percentage, fat mass, lean mass, and FFMI were compared between CD and UC by Student's *t*-test, and comparison between various UC and CD subgroups according to disease extent, location, and behavior; clinical and endoscopic severity; and steroid requirement and disease duration was done by ANNOVA test. *p* value of <0.05 was considered as statistically significant. Data was analyzed by using statistical software SPSS, version 20.

## 3. Results

### 3.1. Baseline Clinical and Demographic Characteristics

A total of 53 patients with ulcerative colitis and 44 patients with Crohn's disease were enrolled in the study (Table 1). Mean age of the UC and CD patients was 33.2 ± 11.2 years and 41.2 ± 15.8 years, respectively. About two-thirds of patients were males in both the groups. Median duration of symptoms in UC and CD patients was 24 (IQR, 12–48) and 36 (IQR, 18–105) months, respectively. Among the UC patients, 5.6% patients had proctitis, 66% had left sided colitis, and 28.3% had pancolitis. Among CD patients, disease location was L1 in 47.7%, L2 in 22.7%, L3 in 25%, and L4 in 22.7% patients; and disease behavior was inflammatory in 45.7%, stricturing in 50% and fistulizing in 4.3% patients. There was significant correlation between subcutaneous fat and fat mass (*r* = 0.9, *p* < 0.001). The treatment details of these patients have been mentioned in Table 1.

### 3.2. Comparison of Body Composition between CD and UC Patients

There was no significant difference in the mean BMI, fat percentage, and fat mass between CD and UC patients (Table 2). The lean mass in CD patients was significantly lower as compared to UC patients (44.2 ± 7.8 kg versus 48.3 ± 8.4 kg, *p* = 0.01) and though not statistically significant, FFMI was also lower in CD as compared to UC patients (17.2 ± 1.8 versus 17.7 ± 1.9, *p* = 0.09).

### 3.3. Effect of Disease Extent, Clinical Disease Severity, Endoscopic Severity, Steroid Requirement, and Disease Duration on Body Composition in Patients of Ulcerative Colitis

#### 3.3.1. Effect of Disease Extent

There was no effect of disease extent on any of the parameters (BMI, fat percentage, fat mass, lean mass, FFMI) (Table 3).

#### 3.3.2. Effect of Clinical Disease Severity

Of 53 patients, 26 were in clinical remission, 17 had mild disease activity, and 10 had moderate to severe disease activity. There was progressive, though not statistically significant, decline in BMI (22.1 ± 4.9 kg/m^2^ versus 20.2 ± 3.2 kg/m^2^ versus 19.9 ± 3.2 kg/m^2^, *p* = 0.23), fat percentage (16.5 ± 10.2% versus 13.9 ± 8.6% versus 9.7 ± 4.9%, *p* = 0.13), and fat mass (10.9 ± 8.9 kg versus 8.1 ± 5.9 kg versus 5.7 ± 3.6 kg, *p* = 0.14) with increase in disease severity. However, there was no effect of disease severity on lean mass and FFMI (Table 3).

#### 3.3.3. Effect of Endoscopic Severity

Of 53 patients, 7 patients had grade I, 37 had grade II, and 7 patients had grade III disease as per Baron's endoscopic severity. Patients having grade II and III disease had significantly lower BMI (20.3 ± 3.8 kg/m^2^ versus 20.8 ± 3 kg/m^2^ versus 24.2 ± 5.3 kg/m^2^, *p* = 0.04) and lesser body fat percentage (12.3 ± 7.9% versus 14.7 ± 5.8% versus 22.6 ± 11.4%, *p* = 0.01) and fat mass (7.4 ± 5.9 kg versus 8.3 ± 3.7 kg versus 16.3 ± 10.8 kg, *p* = 0.004) as compared to patients having grade I score. However, again there was no effect of endoscopic severity on lean mass and FFMI (Table 3).

#### 3.3.4. Effect of Steroid Requirement

There was no effect of steroid requirement on any of the parameters (BMI, fat percentage, fat mass, lean mass, FFMI) (Table 3).

#### 3.3.5. Effect of Disease Duration

Among 53 patients, 14 had a disease duration of <1 year, 30 had a disease duration between 1 and 5 years, and 9 patients had disease duration > 5 years. The BMI (17.9 ± 2.3 kg/m^2^ versus 21.4 ± 2.9 kg/m^2^ versus 21.9 ± 4.8 kg/m^2^, *p* = 0.04) and FFMI (16.1 ± 1.2 versus 18.1 ± 1.7 versus 18.0 ± 2, *p* = 0.004) were significantly lower in patients with disease duration > 5 years as compared to patients with disease duration between 1 and 5 years and disease duration < 1 year. Fat percentage and fat mass also showed a nonsignificant trend towards decline in patients with disease duration > 5 years (Table 3).

### 3.4. Effect of Disease Behavior, Clinical Disease Severity, and Disease Duration on Body Composition in Patients of Crohn's Disease

#### 3.4.1. Effect of Disease Behavior

There was no effect of disease behavior on any of the parameters (BMI, fat percentage, fat mass, lean mass, FFMI) (Table 4).

#### 3.4.2. Effect of Clinical Disease Severity

Of 44 patients, 14 were in clinical remission, 16 had mild disease activity, and 14 had moderate to severe disease activity. Like patients with UC, there was progressive, though not statistically significant, decline in BMI (22.1 ± 4.2 kg/m^2^ versus 19.9 ± 2.3 kg/m^2^ versus 19.7 ± 4.2 kg/m^2^, *p* = 0.18), fat percentage (18.3 ± 10% versus 15.6 ± 8.5% versus 12.9 ± 9.4%, *p* = 0.32), and fat mass (11.2 ± 7 kg versus 7.9 ± 4.4 kg versus 7.2 ± 5.9 kg, *p* = 0.16) with increase in disease severity. However, there was no effect of disease severity on lean mass and FFMI (Table 3).

#### 3.4.3. Effect of Disease Duration

Among 44 patients, 9 had a disease duration of <1 year, 20 had a disease duration between 1 and 5 years, and 15 patients had disease duration > 5 years. The lean mass (40.9 ± 6.5 kg versus 43.6 ± 5.7 kg versus 52.3 ± 8.3 kg, *p* = 0.001) and FFMI (16.3 ± 1.4 versus 17.3 ± 0.9 versus 18.6 ± 2.6, *p* = 0.003) were significantly lower in patients with disease duration between 1 and 5 years and disease duration > 5 years as compared to patients with disease duration < 1 year. BMI and fat mass also showed a nonsignificant trend towards decline in patients with disease duration between 1 and 5 years and disease duration > 5 years (Table 4).

### 3.5. Effect of Disease Behavior, Clinical Disease Severity, and Disease Duration on Abdominal Fat in Patients of Crohn's Disease

#### 3.5.1. Effect of Disease Behavior

There was no difference in visceral and subcutaneous fat area and VF/SC ratio between patients with inflammatory disease versus patients with stricturing/penetrating disease phenotype (Table 5).

#### 3.5.2. Effect of Clinical Disease Severity

There was a statistically nonsignificant trend towards decline in subcutaneous fat area (141.04 ± 77.9 cm^2^ versus 95.9 ± 58.5 cm^2^ versus 86.2 ± 75.5 cm^2^, *p* = 0.10) and increase in VF/SC ratio (0.98 ± 0.4 versus 1.37 ± 0.6 versus 1.53 ± 0.9, *p* = 0.09) with increase in disease severity. However, there was no effect of disease severity on visceral fat area.

#### 3.5.3. Effect of Disease Duration

Like the fat mass, the subcutaneous fat area (88.4 ± 62.8 cm^2^ versus 109.4 ± 71.3 cm^2^ versus 145.3 ± 88.2 cm^2^, *p* = 0.15) was also lower in patients with disease duration between 1 and 5 years and disease duration > 5 years as compared to patients with disease duration less than year. There was no effect of disease duration on visceral fat area and VF/SC ratio.

### 3.6. Effect of Disease Duration on Body Composition in Patients of UC and CD with Disease Activity

Among 27 patients of UC with active disease, 6 had a disease duration of <1 year, 18 had a disease duration between 1 and 5 years, and 3 patients had disease duration > 5 years. Like all patients, the effect of disease duration on BMI, fat percentage, fat mass, and FFMI showed similar trends, although the difference was not significant because of small numbers (Table 6).

Among 30 patients of CD with active disease, 5 had a disease duration of <1 year, 17 had a disease duration between 1 and 5 years, and 8 patients had disease duration > 5 years. Like all patients, the effect of disease duration on lean mass and FFMI showed similar trends (*p* < 0.05) (Table 6).

## 4. Discussion

The present study highlights three major findings: low lean mass and fat-free mass in patients with CD as compared to UC; progressive decline in body fat with increasing disease severity both in UC (clinical as well as endoscopic) and CD (clinical) patients; and decline in fat-free mass with increasing disease duration, again in both UC and CD patients. There was no effect on body composition of disease extent in UC, and disease behavior in CD patients. The main purpose of body composition estimation in these patients is to determine existing differences and set nutritional and therapeutic goals for patients accordingly.

Lower lean mass in CD patients reflects worse nutritional status in CD than UC patients, which could be due to pan-intestinal and transmural involvement in CD resulting in poor oral intake and malabsorption. This finding is consistent with other recent studies from Poland [7] and Hungary [9] and 2 recent systematic reviews in children and adults [24, 25]. This difference in lean mass was evident even when there was no difference in the BMI between the two groups, highlighting the fallacy of BMI in the nutritional assessment of patients with IBD. The fat mass was not different between the two groups and previous studies on this aspect have been inconsistent with some studies showing lower fat mass in CD than UC [7, 8] and others showing similar fat content between the two groups [26].

Correlation of body composition with disease severity has not been well studied, and the results have been inconsistent across literature. In a previous study done from our center, like the present study, the fat mass in patients with active CD was lower than that of patients in remission, whereas there was no difference in the fat-free mass between active CD patients and patients in remission in both the studies [10]. However, unlike the present study, the previous study also included healthy controls, and in comparison to controls, the fat mass was only lower in patients with active CD, whereas fat-free mass was lower in both active and remission groups indicating a poor recovery of lean mass in remission. Similarly, in a study of 57 patients with CD from China [11], patients with active CD had lower BMI than patients with inactive CD and in two other studies from China [11] and Brazil [8], the fat content in patients with active CD was lower than inactive CD. On the other hand, in a systematic review of 19 studies which assessed body composition in adult patients with IBD, there was no consistent association between body composition and disease activity [25]. Endoscopic severity in patients with UC also correlated negatively with fat mass in the present study, and this association was more significant than the association of clinical disease severity, thereby substantiating the association of disease severity and loss of fat mass. This correlation of endoscopic severity with fat mass is a novel concept and has not been shown in previous studies. Increasing disease activity could lead to increased utilization of lipid as a fuel substrate resulting in a reduced fat mass and a preserved fat-free mass [27].

The effect of disease duration on body composition was apparent in both UC and CD patients, but the trend was different. Among patients with UC, the effect of disease duration was seen only after 5 years, as BMI, FFMI, fat mass, and fat percentage were lower only in patients with disease duration > 5 years, whereas, among patients with CD, this effect was seen after 1 year only (lean mass, FFMI, BMI, and fat mass were lower in patients with disease duration > 1 year) indicating that malnutrition sets in early in the disease course of CD as compared to UC, and this can again be explained by the systemic nature of CD. The muscle active cytokines may stimulate protein degradation and inhibit myogenic differentiation and induce myoblast apoptosis, thereby leading to poor lean mass [28]. The corticosteroids administered to these patients may also have a negative effect on the muscle accrual as steroids are well known to enhance adiposity [29]. An inadequate and often self-restricted diet and reduced physical activity may also contribute to a reduced lean mass in these patients.

Visceral fat has been linked to pathogenesis of CD and studies have linked visceral fat with complicated disease behavior [15], postoperative complications [30, 31], and postoperative recurrence in patients with CD [12, 32]. Unlike a previous study we could not correlate visceral fat with disease behavior in our CD patients, and this could possibly be explained by difference in the patient population between the two studies. However, we could link abdominal fat with disease severity as we could demonstrate a nonsignificant negative trend of disease severity with subcutaneous fat (like the fat mass) and a positive trend with VF/SC ratio. Like the fat mass subcutaneous fat also correlated with disease duration with subcutaneous fat being lower in patients with disease duration > 1 year. We could also demonstrate a significant positive correlation between subcutaneous fat on CT and fat mass (assessed by bioimpedance), thereby indicating that in CD patients one can assess body composition with the CT. Similar results were demonstrated in a recent study from Australia where fat-free mass was correlated with skeletal muscle area on CT and fat mass was correlated with subcutaneous fat area on CT [13].

Our study has many limitations. The sample size was small and this could explain nonsignificant trend with many associations, because of type II error. Increasing the sample size could have led to more definite conclusions. We did not include healthy controls in our study, but multiple studies including a previous study from our center have already compared body composition in patients with IBD and controls, so comparison with controls would not have added to the results. Comparison of body composition in the same patients at different stages of endoscopic/clinical activity and at different time points (with respect to disease duration) would also have given better results.

To conclude, Crohn's disease patients are at a higher risk of malnutrition than ulcerative colitis patients, there is a loss of fat mass with increasing disease activity in IBD patients, and their nutritional status deteriorates with increasing disease duration (earlier in CD). Therefore, proper assessment of nutritional status along with proper measures to improve these deficiencies is very important to improve the quality of life in patients with IBD.

## Figures and Tables

**Table 1:** Baseline characteristics of patients of ulcerative colitis and Crohn's disease.

	UC (*N* = 53)	CD (*N* = 44)	*p* value
*Parameters*			

Age in years, mean ± SD	33.2 ± 11.2	41.2 ± 15.8	0.01

Sex, male (*n*, %)	36 (67.9%)	32 (72.7%)	0.61

Duration of symptoms (months), median	24 (12–48)	36 (18–105)	0.15

Haemoglobin, g/dL, mean ± SD	11.8 ± 2.5	10.8 ± 2.9	0.02

Albumin, g/dL, mean ± SD	4.2 ± 0.5	4.0 ± 0.8	0.11

Extent/location of disease	E1: 3 (5.6%) E2: 35 (66%) E3: 15 (28.3%)	L1: 21 (47.7%)	
		L2: 10 (22.7%)	
		L3: 11 (25%)	
		L4: 10 (22.7%)	

Disease behavior		B1: 21 (45.7%)	
		B2: 23 (50%)	
		B3: 2 (4.3%)	

*Treatment received*			

5-ASA	53 (100)	10 (22.7)	

Steroids	16 (30.2)	29 (65.9)	

Immunomodulators	17 (32.1)	26 (40.9)	

Biologics	0 (0)	0 (0)	

E1: proctitis; E2: left sided colitis; E3: pancolitis; L1: terminal ileum ± caecum; L2: colonic; L3: ileocolonic; L4: proximal small intestine; B1: inflammatory; B2: stricturing; B3: penetrating; p: perianal; ASA: aminosalicylic acid.

**Table 2:** Comparison of body composition between patients of Crohn's disease and ulcerative colitis.

Parameters	UC (*N* = 53)	CD (*N* = 44)	*p* value
Body mass index (kg/m^2^), mean ± SD	21.1 ± 4.2	20.5 ± 3.7	0.51
Fat percentage, mean ± SD	14.4 ± 9.1	15.6 ± 9.3	0.52
Fat mass in kg, mean ± SD	9.04 ± 7.5	8.7 ± 5.9	0.81
Lean mass in kg, mean ± SD	48.3 ± 8.4	44.2 ± 7.8	0.01
Fat-free mass index (FFMI), mean ± SD	17.7 ± 1.9	17.2 ± 1.8	0.09

**Table 3:** Effect of disease extent, clinical and endoscopic severity, steroid requirement, and disease duration on body fat and lean mass in patients with ulcerative colitis.

	BMI	Fat percentage	Fat mass in kg	Lean mass in kg	FFMI
*Disease extent*					
E1 (*n* = 3)	21.9 ± 4.5	14.6 ± 9.5	9.4 ± 6.9	48.6 ± 8.4	18.5 ± 2.1
E2 (*n* = 35)	21.3 ± 4.5	15.1 ± 10.1	9.6 ± 8.3	47.3 ± 8.3	17.8 ± 2.1
E3 (*n* = 15)	20.2 ± 3.3	12.8 ± 6.4	7.4 ± 5.1	48.3 ± 8.4	17.5 ± 1.7
*p* *value*	0.64	0.73	0.44	0.77	0.72
*Disease severity*					
Remission (*n* = 26)	22.1 ± 4.9	16.5 ± 10.2	10.9 ± 8.9	49.4 ± 9.3	18.1 ± 2.2
Mild (*n* = 17)	20.2 ± 3.2	13.9 ± 8.6	8.1 ± 5.9	46.5 ± 7.3	17.2 ± 1.9
Moderate to severe (*n* = 10)	19.9 ± 3.2	9.7 ± 4.9	5.7 ± 3.6	48.6 ± 7.8	17.9 ± 1.9
*p* value	0.23	0.13	0.14	0.54	0.36
*Endoscopic severity* ^**∗**^					
Grade I (*n* = 7)	24.2 ± 5.3	22.6 ± 11.4	16.3 ± 10.8	51.9 ± 8.2	18.3 ± 1.6
Grade II (*n* = 37)	20.3 ± 3.8	12.3 ± 7.9	7.4 ± 5.9	47.6 ± 8.5	17.6 ± 2.1
Grade III (*n* = 9)	20.8 ± 3	14.7 ± 5.8	8.3 ± 3.7	47.7 ± 7.9	17.7 ± 1.9
*p* value	0.04	0.01	0.004	0.37	0.62
*Steroid requirement*					
None (*n* = 14)	22.1 ± 5.3	16.7 ± 10.9	11.1 ± 10.1	48.1 ± 9.4	17.9 ± 2.0
1/year (*n* = 15)	20.8 ± 3.6	12.8 ± 7.1	7.5 ± 4.6	48.9 ± 8.1	18.0 ± 1.9
>1/year (*n* = 24)	20.6 ± 3.9	14.1 ± 9.2	8.8 ± 7.1	48.1 ± 8.2	17.5 ± 1.9
	0.59	0.50	0.41	0.95	0.62
*Disease duration (years)*					
<1 year (*n* = 9)	21.4 ± 2.9	14.5 ± 6.1	8.5 ± 4.5	47.6 ± 9.3	18.1 ± 1.7
1–5 years (*n* = 20)	21.9 ± 4.8	15.8 ± 10.6	10.5 ± 8.9	49.4 ± 8.6	18.0 ± 2.0
>5 years (*n* = 15)	17.9 ± 2.3	9.4 ± 6.0	4.9 ± 3.3	45.8 ± 5.5	16.1 ± 1.2
	0.04	0.18	0.13	0.51	0.03

E1: proctitis; E2: left sided colitis; E3: pancolitis; Remission: SCCAI < 3; Mild: SCCAI 3–6; Moderate to severe: SCCAI > 6. ^*∗*^According to Baron's index.

**Table 4:** Effect of disease behavior, clinical severity, and disease duration on body composition in patients with Crohn's disease.

	BMI	Fat percentage	Fat mass in kg	Lean mass in kg	FFMI
*Disease behavior*					
B1 (*n* = 21)	20.6 ± 4.4	14.8 ± 9.7	8.5 ± 6.9	43.8 ± 8.3	17.2 ± 2.0
B2 and B3 (*n* = 22 and 1)	20.5 ± 2.9	16.4 ± 9.1	9.1 ± 5.1	44.4 ± 7.7	16.9 ± 1.7
*p* *value*	0.82	0.54	0.69	0.79	0.91
*Disease severity*					
Remission (*n* = 14)	22.1 ± 4.2	18.3 ± 10.0	11.2 ± 7.0	46.4 ± 7.9	17.7 ± 1.9
Mild (*n* = 16)	19.9 ± 2.3	15.6 ± 8.5	7.9 ± 4.4	41.8 ± 5.7	16.7 ± 1.2
Moderate to severe (*n* = 14)	19.7 ± 4.2	12.9 ± 9.4	7.2 ± 5.9	44.6 ± 9.4	16.9 ± 2.3
*p* value	0.18	0.32	0.16	0.27	0.31
*Disease duration (years)*					
<1 year (*n* = 9)	22.5 ± 5.2	15.6 ± 8.6	10.9 ± 7.6	52.3 ± 8.3	18.6 ± 2.6
1–5 years (*n* = 20)	19.4 ± 3.3	15.1 ± 10.0	7.8 ± 5.8	40.9 ± 6.5	16.3 ± 1.4
>5 years (*n* = 15)	20.8 ± 2.5	16.2 ± 9.4	8.6 ± 5.1	43.6 ± 5.7	17.3 ± 0.9
*p* value	0.11	0.95	0.44	0.001	0.003

B1: inflammatory; B2: stricturing; B3: penetrating; Remission: CDAI < 150; Mild: CDAI: 150–220; Moderate to severe: CDAI > 220.

**Table 5:** Effect of disease behavior, clinical severity, and disease duration on visceral and subcutaneous fat area and visceral to subcutaneous fat ratio (VF/SC).

	Visceral fat area	Subcutaneous fat area	VF/SC ratio
*Disease behavior*			
B1 (*n* = 21)	93.1 ± 59.2	105.8 ± 79.5	1.32 ± 0.8
B2 and B3 (*n* = 22 and 1)	114.9 ± 79.2	108.4 ± 68.1	1.28 ± 0.7
*p* value	0.31	0.91	0.90
*Disease severity*			
Remission (*n* = 14)	115.1 ± 54.1	141.04 ± 77.9	0.98 ± 0.4
Mild (*n* = 16)	97.6 ± 54.9	95.9 ± 58.5	1.37 ± 0.6
Moderate to severe (*n* = 14)	101.8 ± 99.2	86.2 ± 75.5	1.53 ± 0.9
*p* value	0.79	0.10	0.09
*Disease duration (years)*			
<1 year (*n* = 9)	106.5 ± 60.8	145.3 ± 88.2	1.09 ± 0.5
1–5 years (*n* = 20)	93.6 ± 84.6	88.4 ± 62.8	1.36 ± 0.8
>5 years (*n* = 15)	117.8 ± 55.4	109.4 ± 71.3	1.33 ± 0.6
*p* value	0.61	0.15	0.63

B1: inflammatory; B2: stricturing; B3: penetrating; Remission: CDAI < 150; Mild: CDAI: 150–220; Moderate to severe: CDAI > 220.

**Table 6:** Effect of disease duration on body composition in patients of ulcerative colitis (UC) and Crohn's disease (CD) with active disease (excluding patients in remission).

Disease duration (years)	BMI	Fat percentage	Fat mass in kg	Lean mass in kg	FFMI
*Ulcerative colitis *(*n* = 27)					
<1 year (*n* = 6)	20.9 ± 2.8	13.9 ± 6.7	7.6 ± 4.3	45.0 ± 6.5	17.4 ± 1.1
1–5 years (*n* = 18)	20.3 ± 3.2	12.8 ± 8.1	7.7 ± 5.7	47.6 ± 8.1	16.9 ± 1.8
>5 years (*n* = 3)	17.1 ± 2.2	6.1 ± 3.9	3.4 ± 2.7	49.5 ± 5.2	15.5 ± 1.3
*p* value	0.19	0.31	0.43	0.67	0.28
*Crohn's disease *(*n* = 30)					
<1 year (*n* = 5)	20.8 ± 5.6	11.4 ± 8.6	7.7 ± 7.6	50.7 ± 8.4	17.6 ± 2.7
1–5 years (*n* = 17)	19.4 ± 2.8	15.6 ± 9.2	7.8 ± 4.9	40.5 ± 7.0	15.7 ± 1.4
>5 years (*n* = 8)	20.2 ± 2.5	13.5 ± 8.9	7.0 ± 4.6	44.0 ± 5.6	16.8 ± 1.0
*p* value	0.65	0.62	0.95	0.03	0.05
